# Burden of health behaviours and socioeconomic position on health care expenditure in Ontario

**DOI:** 10.12688/f1000research.18205.2

**Published:** 2019-10-16

**Authors:** Douglas G. Manuel, Carol Bennett, Richard Perez, Andrew S. Wilton, Adrian Rohit Dass, Audrey Laporte, David A. Henry

**Affiliations:** 1 Ottawa Hospital Research Institute, Ottawa, Canada; 2 ICES, Toronto and Ottawa, Canada; 3 Statistics Canada, Ottawa, Canada; 4 Department of Family Medicine, University of Ottawa, Ottawa, Canada; 5 School of Epidemiology and Public Health, University of Ottawa, Ottawa, Canada; 6 Bruyère Research Institute, Ottawa, Canada; 7 Institute of Health Policy, Management and Evaluation, University of Toronto, Toronto, Canada; 8 Centre for Research in Evidence-Based Practice, Bond University, Gold Coast, Australia

**Keywords:** Burden, smoking, alcohol, diet, physical activity, healthcare cost

## Abstract

**Background: **Smoking, unhealthy alcohol consumption, poor diet and physical inactivity are leading risk factors for morbidity and mortality, and contribute substantially to overall healthcare costs. The availability of health surveys linked to health care provides population-based estimates of direct healthcare costs. We estimated health behaviour and socioeconomic-attribute healthcare costs, and how these have changed during a period when government policies have aimed to reduce their burden.

**Methods:** The Ontario samples of the Canadian Community Health Surveys (conducted in 2003, 2005, and 2007-2008) were linked at the individual level to all records of health care use of publicly funded healthcare. Generalized linear models were estimated with a negative binomial distribution to ascertain the relationship of health behaviours and socioeconomic risk factors on health care costs. The multivariable cost model was applied to unlinked, Ontario CCHS samples for each year from 2004 to 2013 to examine the evolution of health behaviour and socioeconomic-attributable direct health care expenditures over a 10-year period.

**Results:** We included 80,749 respondents, aged 25 years and older, and 312,952 person-years of follow-up. The cost model was applied to 200,324 respondents aged 25 years and older (CCHS 2004 to 2013). During the 10-year period from 2004 to 2013, smoking, unhealthy alcohol consumption, poor diet and physical inactivity attributed to 22% of Ontario’s direct health care costs. Ontarians in the most disadvantaged socioeconomic position contributed to 15% of the province’s direct health care costs. Combined, these health behaviour and socioeconomic risk factors were associated with 34% ($134 billion) of direct health care costs (2004 to 2013). Over this time period, we estimated a 1.9% reduction in health care expenditure ($5.0 billion) attributable to improvements in some health behaviours, most importantly reduced rates of smoking.

**Conclusions:** Adverse health behaviours and socioeconomic position cause a large direct health care system cost burden.

## Introduction

Smoking, unhealthy alcohol consumption, poor diet and physical inactivity are leading risk factors for morbidity and mortality worldwide ^1^. Despite this knowledge, prevalence of these risk factors remains high and reduction efforts may be hindered by failure to understand the full human and cost burdens these risk factors impose on societies ^2^. In an era of increasing health care expenditure most political focus has been on payments for services and the growing impacts of diagnostic and therapeutic technologies. There is also a need to consider costs resulting from upstream health behaviours, and how these have changed over time, to help prioritize public health strategies and support public health decision makers. These relationships are likely to be complex because of some conflicting trends in the prevalence of health behaviours ^3, 4^.

While health behaviours have a leading role in morbidity and mortality, it is also recognized that there is uneven distribution of health across socioeconomic position (i.e., social and economic factors that influence what position individuals hold within the structure of a society ^5^). Canadian research indicates that individuals with lower socioeconomic position tend to be less healthy than those who enjoy greater educational, income and occupational advantages ^6– 9^. As with health behaviour-attributable health care use, evidence of the economic cost of these health disparities helps us understand the health and financial benefits of reducing the gap ^6^.

We sought to estimate the economic burden attributable to four health behaviour risk factors (smoking, unhealthy alcohol consumption, poor diet and physical inactivity), how these have changed over time, and how they interact with socioeconomic position. The study had three objectives: 1) to examine the direct healthcare costs associated with smoking, unhealthy alcohol consumption, poor diet and physical inactivity; 2) to examine the change in direct health care costs as a consequence of changes, over time, in these health behaviours; and, 3) to examine the direct health care costs associated with socioeconomic position (i.e., education, family income, home ownership, and neighbourhood deprivation).

Past studies typically infer health care costs indirectly—where aggregate health care expenditure data is categorized by disease, for example. We used unique Canadian data that individually link respondents from large repeated population health surveys to comprehensive health care utilisation and cost data covering hospital and primary care sectors in Ontario. These data provide, to our knowledge, the largest and most complete population-based examination of the relationship between health behaviours and direct public healthcare costs. We believe this is the first study to measure directly how changes in health behaviours result in changes in health care use. The linked data also provide the means to assess the degree to which health costs are associated with socioeconomic inequalities.

## Methods

### Ethical approval

This study was approved by the Ottawa Health Science Network Research Ethics Board. Datasets were linked using unique encoded identifiers and analysed at ICES. ICES is an independent, non-profit research institute whose legal status under Ontario’s health information privacy law allows it to collect and analyse health care and demographic data, without consent, for health system evaluation and improvement.

### Study cohorts

We used the Ontario sample drawn from a national population health survey—the Canadian Community Health Survey (CCHS), conducted in 2003, 2005, 2007-2008, 2009-2010, 2011-2012, and 2013-2014 to develop linked and unlinked study cohorts. The CCHS is a cross-sectional survey conducted by Statistics Canada that collects data related to health determinants, health status and health care use—details of data collected and used in the analyses are provided below. The survey employs a complex multistage sampling strategy to randomly select households in each health region. Details of the survey methodology have previously been published ^10^. A weight, which reflects the number of individuals represented in the target population, is assigned to each respondent; the target population includes individuals aged 12 years and older living in Canada’s ten provinces and three territories. Individuals living on First Nation Reserves, institutionalized residents, full-time members of the Canadian Forces and residents of certain remote areas are excluded from the survey’s sampling frame.

**Figure 1.**
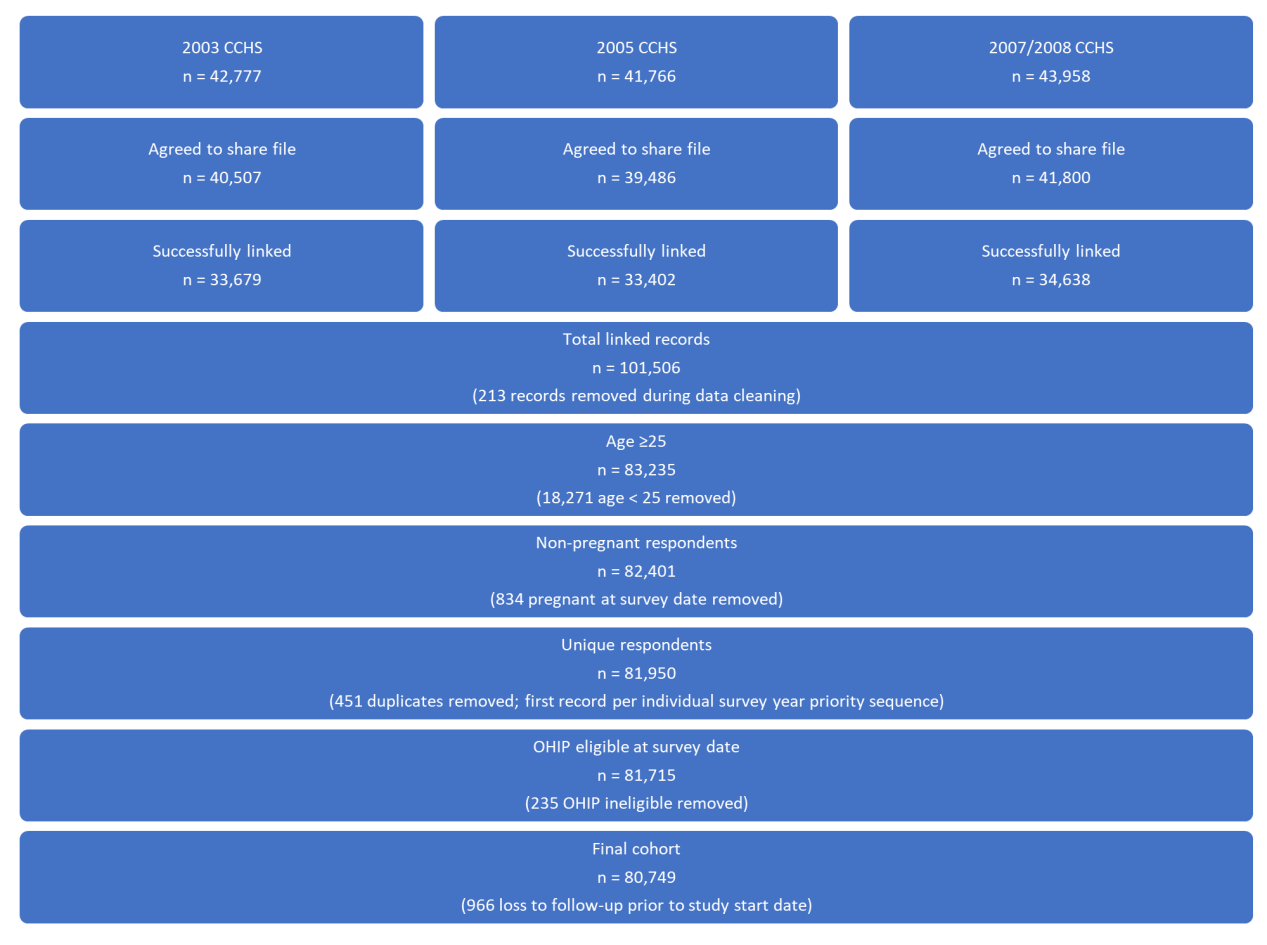
Creation of study cohort for multivariable cost model. CCHS, Canadian Community Health Survey; OHIP, Ontario Health Insurance Program.

For the linked study cohort, the Ontario sample of the 2003, 2005, and 2007-2008 CCHS cycles provided 128,501 valid interviews. Of these respondents, a subset agreed to share and link their interview information and 101,506 were successfully linked to their provincial health card number using a deterministic and probabilistic algorithm. We included respondents aged 25 years and older if they were eligible for provincially funded health care and not pregnant at the time of survey administration. For individuals with multiple interviews, only the earliest interview was included. We excluded individuals who were lost to follow-up in the first year following their interview (i.e., they were not available at the beginning of the study). This resulted in a final cohort of 80,749 unique Ontario respondents (see Figure 1).

The Ontario sample of all six CCHS waves provided 200,324 valid respondents aged 25 years and older for the unlinked study cohort. This cohort was used to estimate direct health care costs by applying the multivariable model that was derived using the linked cohort CCHS data (see Model Development).

### Behavioural and other risk factors for health care use

The CCHS waves were used to examine the following risk factors for their association with health care use: age, sex, four health behaviours (smoking, alcohol consumption, diet and physical activity), sociodemographic factors (immigrant status, education level, urban dwelling, neighbourhood deprivation, household income, home ownership, marital status), self-perceived stress, preventive health behaviour (flu vaccination), and health status indicators (body mass index, hypertension, diabetes, heart disease, cancer, history of stroke, dementia, and extent of difficulty in performing basic tasks or participating in activities).

Smoking behaviour was described by combining separate questions about smoking status, daily cigarette consumption, and past smoking behaviour. We categorized current smokers and former smokers as heavy or light (see Table 1). Alcohol drinking behaviour was specified as heavy, moderate and light/non using cut-points for daily alcohol consumption and the presence of bingeing behaviour (see Table 1). Physical activity was included as the average daily energy expended during leisure time activities by the respondent. The energy expenditure was calculated using the frequency and duration per session of the physical activity as well as the metabolic equivalent of task (MET) value of the activity. The MET is a value of metabolic energy cost expressed as a multiple of the resting metabolic rate and tends to be expressed in three intensity levels (i.e., low, medium, high). The CCHS questions did not ask the respondent to specify the intensity level of their activities. We used the MET values adopted correspond to the low intensity value of each activity—an approach adopted from the Canadian Fitness and Lifestyle Research Institute that responds to the tendency of individuals to overestimate the intensity, frequency and duration of their activities. Using the same criteria as the Ontario Health Study and the Campbell Survey on Well-Being in Canada, physical activity was categorized as active, moderately active, or inactive (see Table 1). Diet was included using an index (the Perez Diet Score) that considers the possibility that different dietary components can be protective (fruit and vegetable and carrot consumption) or harmful (high potato or fruit juice consumption) ^11^. The index was categorized into three groups (see Table 1).

**Table 1.** Definitions of behavioural health risks.

Behaviour	Category *	Definition
Smoking	**Heavy smoker**	**Current daily smoker (≥20 cigarettes/day)**
	Light smoker	Current daily smoker (<20 cigarettes/day) or current occasional smoker with ≥100 lifetime cigarettes
	Former heavy smoker	Former daily smoker (≥20 cigarettes/day)
	Former light smoker	Former daily smoker (<20 cigarettes/day) or former occasional smoker with ≥100 lifetime cigarettes
	*Non-smoker*	*Never smoker or occasional smoker <100 lifetime cigarettes*
Alcohol	**Heavy drinker**	**Bingeing ‡ or >21 (men) or >14 (women) drinks/week**
	*Moderate drinker*	*≤21 (men) or ≤14 (women) drinks/week with no bingeing*
	Non-drinker	No alcohol consumption in the last 12 months
Diet	**Poor diet**	**Index score † 0 to <2.5**
	Fair diet	Index score 2.5 to <5
	*Adequate diet*	*Index score 5 to 10*
Physical activity	**Inactive**	**0 to <1.5 MET-hours/day**
	Moderately active	1.5 to <3 MET-hours/day
	*Active*	*≥3 MET-hours/day*

*Highest risk levels are in bold and lowest risk levels (reference group) are in italics. ^‡^Bingeing: five or more drinks on any day in the previous week or weekly bingeing behaviour in the previous month. †Index score: the healthiness of a diet based on consumption of fruit and vegetables. Individuals start with 2 points and achieve up to 8 additional points for each average daily serving of fruits and vegetables (maximum score = 10). Points are deducted for daily fruit juice servings exceeding 1 (-2 points), no carrot consumption (-2 points), or daily potato consumption exceeding 1 serving for males and 0.7 servings for females (-2 points). Scores that result in negative values after deductions are recoded to zero, resulting in a final range of 0 to 10 for the index. MET, metabolic equivalent of task (a measure of calories burned by type, duration and frequency of physical activity).

All sociodemographic and health status indicators were based on the self-reported responses and are presented in Table 2, however, our area-based measure of deprivation requires additional detail. Neighbourhood deprivation was developed using the Deprivation Index originally published by Pampalon and Raymond ^12^. The index, which serves as a proxy for individual-level measures, categorizes the smallest geo-statistical units of the Canadian census (dissemination areas) into two sets of quintile groups. The first quintile group, for material components of deprivation, is based on average income, percent without high school graduation, and the employment ratio. The second quintile group, for social components of deprivation, is based on percent of single-parent families, percent of people living alone, and percent of people divorced, widowed or separated) ^13^. In each quintile group, Q1 represents the 20% least deprived and Q5 represents the 20% most deprived. These quintiles are cross tabulated to create 25 distinct cells. Dissemination areas with material and social combinations in the first and second quintiles (four cells) were categorized as having low neighbourhood deprivation. Dissemination areas with material and social combinations in the fourth and fifth quintiles (four cells) were categorized as having high neighbourhood deprivation. All other dissemination areas were categorized as having moderate neighbourhood deprivation. 

**Table 2.** Baseline description of the study cohorts.

	Male cohort			Female cohort		
	Survey sample * %	Person- years	Represented population ^‡^ %	Survey sample ^*^ %	Person- years	Represented population ‡ %
	(N=36,807)		(N=3,962,088)	(N=43,942)		(N=4,131,570)
**Age group (years)**						
25 to 29	7.4	10,668	9.1	7.3	12,642	8.8
30 to 34	9.0	13,001	10.0	8.5	14,714	9.1
35 to 39	10.3	14,957	11.5	9.1	15,888	11.5
40 to 44	11.1	16,183	14.5	8.8	15,326	12.5
45 to 49	9.0	13,059	11.5	8.1	14,149	11.6
50 to 54	9.6	13,847	10.2	9.4	16,256	10.2
55 to 59	10.0	14,420	9.6	10.3	17,760	9.1
60 to 64	9.0	12,897	7.2	9.0	15,451	7.2
65 to 69	7.9	11,152	5.6	8.0	13,568	6.2
70 to 74	6.8	9,408	4.6	7.5	12,650	5.0
75 to 79	5.3	7,004	3.4	6.5	10,654	4.2
80 to 84	3.1	3,907	2.0	4.8	7,575	2.8
85 to 89	1.2	1,356	0.7	2.1	3,257	1.3
90+	0.3	320	0.2	0.7	883	0.4
***Health Behaviours***						
**Smoking status**						
Heavy smoker	10.7	15,116	9.2	5.8	9,828	4.8
Light smoker	14.5	20,642	15.4	14.8	25,234	13.8
Former heavy smoker	20.0	27,998	16.0	9.5	16,010	7.7
Former light smoker	17.4	24,531	16.8	18.0	30,678	16.3
Non-smoker	36.5	52,432	41.8	51.0	87,361	56.6
Missing	1.1	1,460	0.9	1.0	1,661	0.8
**Alcohol consumption**						
Heavy drinker	12.3	17,695	10.9	3.4	5,762	3.3
Moderate drinker	70.7	100,787	71.8	72.2	124,162	70.0
Non-drinker	15.1	20,940	15.3	23.3	38,974	25.6
Missing	2.0	2,758	2.0	1.1	1,876	1.1
**Physical activity**						
Inactive	46.5	65,794	47.8	52.8	89,455	54.3
Moderately active	25.2	36,032	24.3	25.6	44,103	24.5
Active	26.1	37,473	25.1	20.8	35,913	19.7
Missing	2.2	2,880	2.8	0.9	1,302	1.5
**Diet**						
Poor diet	15.0	21,400	14.2	8.6	14,632	8.3
Fair diet	41.7	59,340	40.3	30.0	50,933	29.2
Adequate diet	38.6	55,167	40.7	58.2	99,981	59.0
Missing	4.7	6,272	4.8	3.3	5,227	3.6
***Sociodemographic*** ***Indicators***						
**Immigrant status**						
Immigrant	21.8	30,837	33.5	21.5	36,779	33.9
Non-immigrant	78.1	111,177	66.2	78.3	133,765	65.8
Missing	0.1	165	0.3	0.1	228	0.4
**Ethnicity**						
White	88.9	126,274	78.9	89.7	152,990	79.3
Non-white	10.7	15,357	20.5	10.0	17,183	20.1
Missing	0.4	548	0.6	0.4	600	0.5
**Education**						
Less than high school	18.9	25,984	14.8	20.2	33,702	16.5
High school graduate	22.7	32,530	22.2	25.0	42,730	24.9
Post-secondary graduate	57.5	82,404	61.8	54.1	93,212	57.7
Missing	0.9	1,262	1.2	0.7	1,129	0.9
**Marital status**						
Married/common-law	67.8	96,810	76.8	57.7	99,508	68.8
Other	32.2	45,306	23.2	42.3	71,197	31.2
Missing	0.0	64	0.0	0.0	68	0.0
**Residence ownership**						
Yes	79.3	113,229	79.9	75.9	130,283	77.8
No	20.5	28,730	19.7	23.9	40,246	21.9
Missing	0.2	221	0.3	0.1	244	0.3
**Household income ($)**						
0 to 29,999	16.6	22,903	11.0	25.9	43,299	16.4
30,000 to 79,999	45.5	64,687	40.1	41.6	71,561	39.3
80,000+	32.2	46,643	40.3	23.5	40,774	31.0
Missing	5.7	7,946	8.6	9.0	15,140	13.3
**Preventive healthcare**						
**Flu shot**						
Yes	61.6	87,077	58.0	68.6	116,714	63.4
No	36.0	51,907	38.9	30.5	52,701	35.0
Missing	2.5	3,195	3.1	0.9	1,358	1.5
**Geography**						
**Urban**						
No	22.3	31,709	14.9	21.0	36,089	14.3
Yes	77.7	110,471	85.1	79.0	134,684	85.7
**Neighbourhood** **deprivation**						
High	15.1	21,394	11.8	16.3	27,519	12.6
Moderate	62.3	88,446	60.8	62.3	106,413	61.2
Low	20.6	29,532	25.3	19.3	33,198	24.2
Missing	2.0	2,807	2.1	2.2	3,643	2.0
**General health indicators**						
**Self-perceived stress**						
Quite a bit or extremely stressful	20.2	28,881	23.0	21.5	36,892	24.4
At most, a bit stressful	79.5	112,917	76.7	78.2	133,339	75.4
Missing	0.3	382	0.3	0.3	542	0.3
**Body mass index**						
Underweight	0.7	926	0.8	2.7	4,428	3.2
Normal	43.5	62,221	43.3	29.9	51,187	28.3
Overweight	15.6	22,224	13.9	12.5	21,474	10.9
Obese	4.8	6,884	4.0	6.3	10,740	5.3
Morbidly Obese	34.2	48,428	36.5	45.5	77,566	48.5
Missing	1.2	1,497	1.5	3.2	5,378	3.9
**Indicators of illness**						
**Hypertension**						
Yes	22.7	31,629	18.9	25.7	43,146	20.5
No	77.0	110,133	80.8	74.2	127,408	79.4
Missing	0.3	417	0.3	0.1	218	0.1
**Diabetes**						
Yes	8.7	11,757	7.2	7.5	12,319	6.2
No	91.3	130,305	92.7	92.5	158,364	93.8
Missing	0.1	118	0.1	0.1	90	0.0
**Heart disease**						
Yes	9.4	12,559	6.8	7.9	12,819	5.5
No	90.4	129,348	93.0	91.9	157,654	94.3
Missing	0.2	272	0.1	0.2	299	0.2
**Cancer**						
Yes	2.9	3,754	2.1	2.6	4,199	2.0
No	97.0	138,284	97.9	97.3	166,374	97.9
Missing	0.1	141	0.1	0.1	200	0.1
**Stroke**						
Yes	2.0	2,552	1.4	1.8	2,796	1.4
No	98.0	139,540	98.6	98.1	167,826	98.6
Missing	0.1	88	0.1	0.1	151	0.0
**Dementia**						
Yes	0.6	645	0.5	0.4	629	0.5
No	99.4	141,440	99.5	99.5	170,034	99.4
Missing	0.1	94	0.1	0.1	109	0.1
**Fragility**						
Help with basic tasks	6.6	8,570	5.4	13.6	22,096	11.4
Limitation due to health	21.7	30,749	18.8	20.3	34,852	17.8
No limitations	71.4	102,527	75.5	65.8	113,449	70.6
Missing	0.3	334	0.3	0.2	376	0.2

*Data source: Canadian Community Health Survey (CCHS) 2.1, 3.1 and 4.1 (2003, 2005 and 2007/08). ^‡^Population estimated using the CCHS sampling weights.

### Public health care spending data

Canada’s health care system is publicly funded and built on the principal of universal coverage for medically necessary health care services. The federal government sets national principals for the health care system and provides transfer payments to the provinces and territories who, in turn, administer and deliver health care services. While they are expected to meet the national principals, it is up to the individual provincial and territorial health insurance plans to determine which services are medically necessary for health insurance purposes and to decide whether supplementary benefits, like dental care, home care, long-term care, and drug coverage, are covered. Those who do not qualify for supplementary benefits under government plans pay for these services (ether through out-of-pocket payments of through private insurance plans). Health expenditures vary across the provinces and territories —due, in part, to differences in the services that each province and territory covers as well as to sociodemographic differences. Nationally, approximately 70% if healthcare expenditures is publicly funded ^14^. In Ontario, the focus of the study, out-patient prescription drugs are, for the most part, not publicly funded for people less than age 65 years unless they receive low-income social assistance (see next section).

We examined publicly funded person-level health care costs across three sectors in Ontario: 1) hospital care (inpatient hospitalizations, same day surgeries, emergency department visits, rehabilitation hospitals, and complex continuing care centres); 2) drugs (for Ontarians age 65 and older and Ontarians receiving social assistance, Ontario Drug Benefit costs were captured); and 3) community care (primary care billings, specialist billings, lab billings, capitation services, and home care services).

Costs to operate the provincial health care system (e.g., health ministry administrative costs) and capital costs for large scale projects (e.g., building new hospitals) are not reflected in the person-level costs. To account for these exclusions in the health care cost analysis, we obtained annual total health care expenditures from publicly available Ontario Ministry of Finance records (fiscal years 2003 to 2013, where fiscal year is April 1 to March 31), the Canadian Institute for Heath Information’s *National Health Expenditure Trends* publication, and the MOHLTC’s *Report Card for the Ontario Drug Benefit Program* publications ^15– 27^. The expenditures from each year were categorized into our three health care sectors and expenditures that did not correspond with any of the three sectors were assigned to an ‘other’ category.

All costs are expressed in 2014 Canadian dollars with past costs inflated using the annual general Consumer Price Index reported by Statistics Canada.

### Model development

To develop multivariable cost models, Ontario respondents to the 2003, 2005 and 2007-2008 cycles of CCHS were linked, at the individual level, to all records of health care use that were paid for by the Ontario Ministry of Health and Long-Term Care (MOHLTC). The cost associated with each record was estimated using costing methods developed for Ontario health administrative data ^28^. Briefly, a payer (the MOHLTC) cost perspective was taken, using person-level health care utilization and per-use fee information or budgetary data. Cost information for sectors (i.e., acute hospitalization, same day surgery, emergency department, inpatient rehabilitation, and complex continuing care) that are funded using global budgets (e.g., by institution) are determined using a top-down approach through case-mix methodology. Sectors that have fee payments associated with each use (e.g., prescription drugs, physician services, and home care) have costs estimated directly.

**Figure 2.**
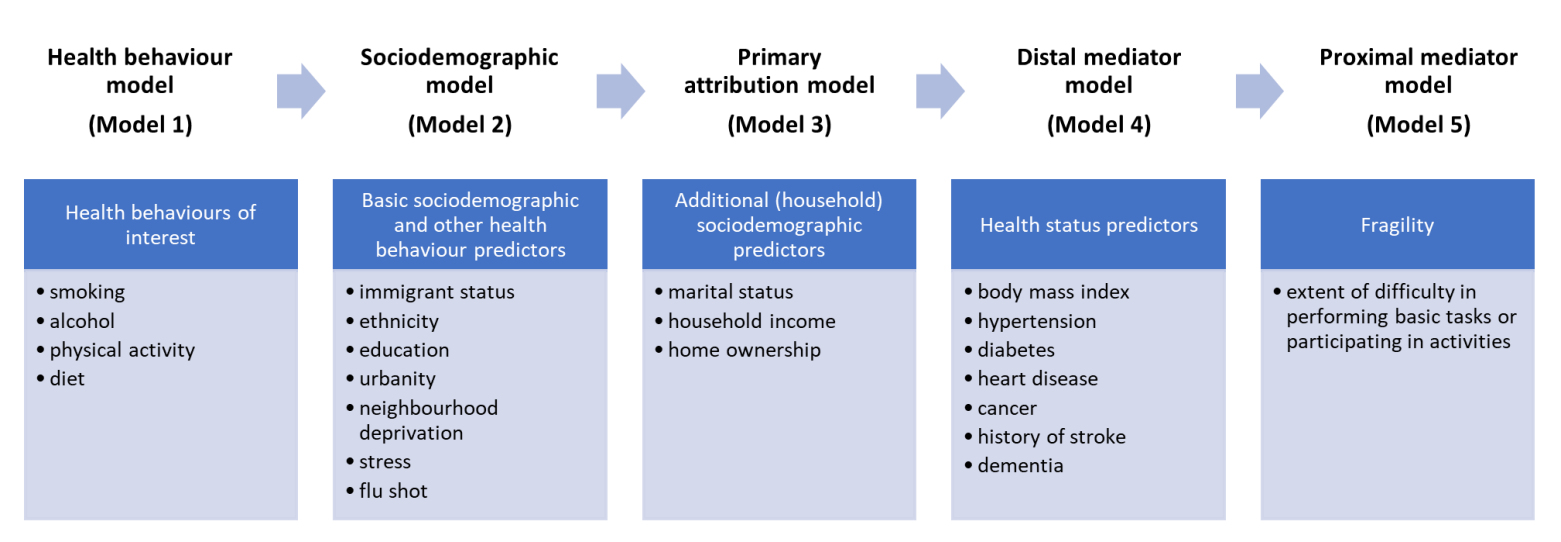
Stepped approach to model building—consideration of risk factors leading to healthcare use and costs.

Beginning one year after survey administration, CCHS respondents were followed for a four-year period (between 2004 and 2013) to develop multivariable models estimating the effect of health behaviours on health care costs. The four-year time frame enabled equal follow-up time for each CCHS cycle within the available linked data at the time of analysis. We used a generalized linear model with a negative binomial distribution and an offset to account for variation in follow-up times to create separate, sex-specific models for each of the health care sectors: hospital care, drugs, and community care. To assess confounding and mediation, we use a pre-specified, stepwise modelling approach. We started with a *health behaviour model* followed by a basic *sociodemographic model*, a *primary attribution model* that adjusted for additional sociodemographic risk factors, a *distal mediator model* that included health status indicators, and a *proximal mediator model* that included a measure of fragility (see Figure 2). This stepwise analysis resulted in 15 models for each sex.

The model building approach sought to address three issues in assessing the contribution of health behaviours to health care costs. First, we were interested in having appropriate adjustment for other risk factors for increased costs that are correlated with health behaviours (e.g., age and sociodemographic risk factors). Second, we were attentive to risk factors that may mediate the relationship between health behaviours and health care costs (e.g., body mass index or hypertension). Our concern was that their inclusion in the model could inappropriately attenuate the risk from health behaviours. Third, we considered pre-existing illness that may have led to health behaviour change. For example, as people become ill and frail they may become less physically active; in such a situation, physical inactivity may be associated with increased health care cost that is more appropriately identified as illness-associated inactivity. The estimate derived from Model 3 (primary attribution model) was assumed to be our most accurate and appropriate estimate of the attributable burden due to health behaviours and socioeconomic position.

### Estimating population attributable fractions of health care costs

We calculated the proportion of the health care costs that can be attributed to health behaviours—the population attributable fraction—for fiscal years 2003 to 2013, for each health care sector. Using the unlinked CCHS cohort, we estimated annual population attributable fractions using each CCHS cycle and for years between CCHS cycles, by averaging the population attributable fractions from the preceding and succeeding year.

We used a factor-deleted approach to calculate population attributable fractions that involved three steps. In the first step, we estimated expected annual population health care costs for a specific sector by applying the corresponding sector-specific primary attribution models to the weighted CCHS cycle. In the second step, we repeated the calculation after recoding each respondent’s health behaviour to the counterfactual reference or “no exposure” category. For example, taking the weighted cohort, we estimated hospital costs using all smoking exposures (i.e., current, former, and non-smokers) and re-estimated hospital costs assuming all current and former smokers were non-smokers. The difference between the two calculations was an estimate of the annual contribution of smoking to hospital costs. In the final step, we divided this difference by the original population estimate (from the first step) to produce a population attributable fraction. In our example, this would be the population attributable fraction of hospital costs associated with smoking. Health sector specific population attributable fractions were calculated for each health behaviour, and the combination of health behaviours.

The same analysis was performed for different socioeconomic groups defined by education level, family income, home ownership, and neighbourhood deprivation. The equity gap in health care use was defined as the difference in cost between socioeconomic groups. Meaning, we calculated expected health care costs if all Ontarians were at the socioeconomic category with the lowest health care costs (that is, those with post-secondary graduation, household income of $80,000 or more, residence owned by a household member and low neighbourhood deprivation).

### Estimating the health care cost burden of health behaviours and socioeconomic position in Ontario

We calculated annual estimates of costs attributable to health behaviours and socioeconomic position (fiscal years 2004 to 2013) by applying the sector-specific population attributable fractions to the annual public health care expenditures and summing the health care sector results together. The population attributable fraction for community care was applied to ‘other’ health care costs.

### Estimating costs attributable to changes in health behaviours

The change in health care costs attributable to the change in health behaviours was estimated annually for fiscal years 2004 to 2013. A baseline population attributable fraction for total health care costs associated with all health behaviours was estimated for fiscal year 2003 using the previously described methods for population attributable fractions and attributable costs. The overall health care budget was estimated annually over the subsequent decade, assuming that health behaviours in 2003 remained constant (e.g., the baseline population attributable fraction did not change over time). The difference between the counterfactual health care budget and the actual health care budget in each year provided an annual estimate of the change in health care costs attributable to changes in health behaviours.

### Sensitivity analysis

We performed three sets of sensitivity analyses. First, the estimates derived from Model 1 (simply age and health behaviours) and Model 5 (the over-adjusted model) of our stepwise approach to assess confounding and mediation ( Figure 2) were used as upper and lower bounds of uncertainty around our primary attribution model. We did not create a model with simply age and socioeconomic position.

For our second sensitivity analysis, we compared age-standardized cost ratios after excluding the top 5% of health care users to assess the possibility of overly influential respondents. The use of health care varies considerably between people, particularly for hospital care and other speciality services. Only a small proportion of people are hospitalized and, of those hospitalized, a small proportion has multiple admissions and complicated long hospital stays. The skewed distribution of health care services has potential to distort the attributable health care expenditure analysis because a small proportion of CCHS respondents may have a strong influence on overall or total population estimates.

Third, we replicated analyses using an inverse propensity-weighted model to assess robustness of the health care cost ratios attributable to smoking. The inverse propensity-weighted model, a complimentary approach to the generalized linear model, is an alternative approach to adjust cost ratios for multivariable risk factors. The propensity score is defined as the probability of treatment assignment (e.g., non-smoker versus heavy smoker) conditional on observed baseline covariates ^29– 31^. Weighting subjects by the inverse probability of treatment received allows one to obtain unbiased estimates of average treatment effects ^32^. Our inverse propensity-weighted analyses included several covariates in addition to all those used in the multivariable analyses (i.e., the primary attribution model): household type, highest level of household education, main source of household income, labour force participation, sense of belonging to the community, regional health authority, and survey cycle.

## Results

The population attributable fractions for the four behavioural risks were calculated using responses from 80,749 Ontarians surveyed between 2003 and 2008. In total, there were 312,952 person-years of follow-up. Characteristics of the study cohort are presented in Table 2.

### Health behaviour attributable healthcare use

**Figure 3.**
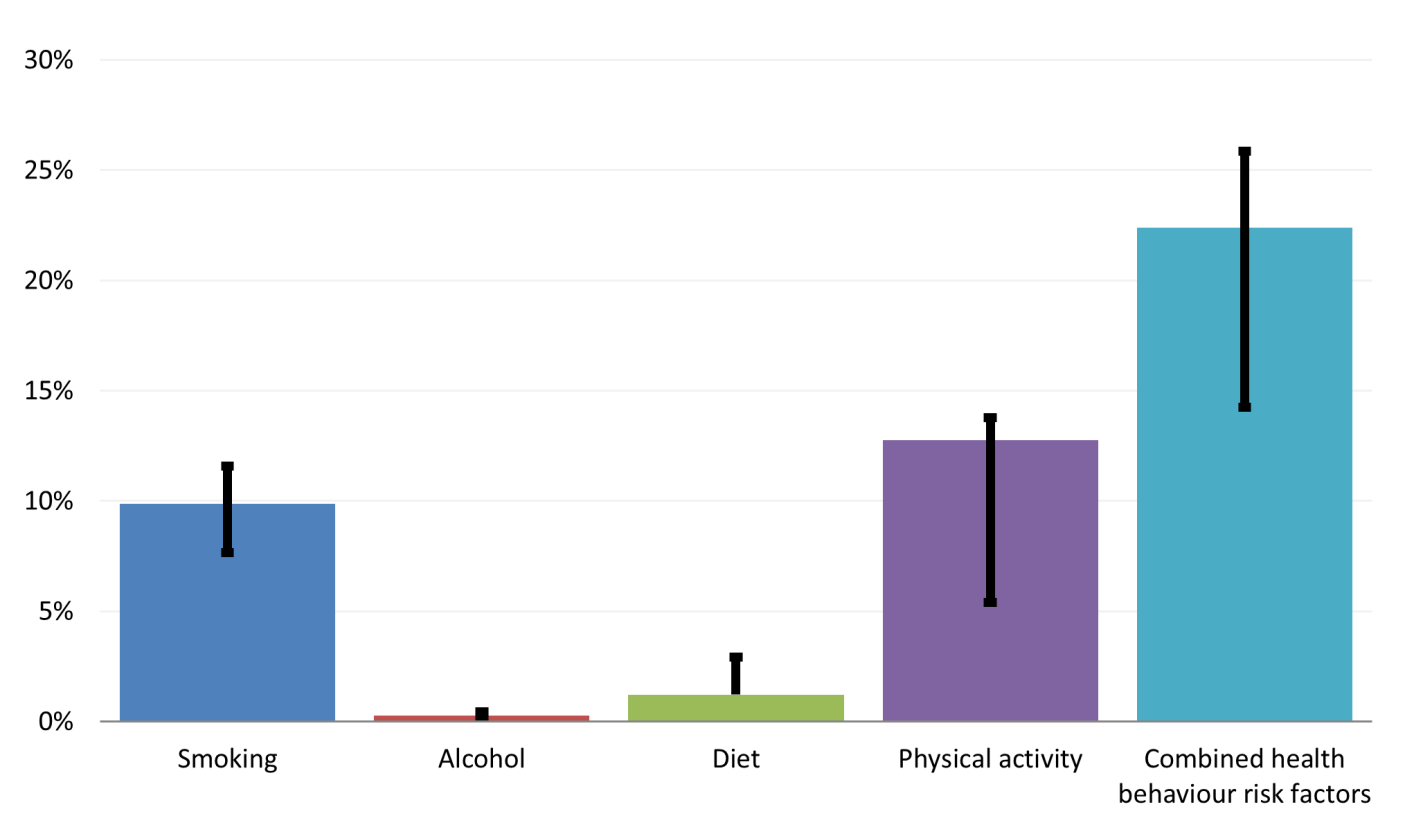
Proportion of health care costs attributed to selected health behaviour risk factors for Ontarians aged 25 and older, 2004 to 2013. Error bars represent high and low boundaries on burden estimates.

From fiscal years 2004 to 2013, 22% of Ontario’s health care costs could be attributed to the four health behaviour risk factors ( Figure 3). Physical activity had the largest attribution (13%), followed by smoking (10%). However, uncertainty for the burden estimates (i.e., the high and low boundaries from our sensitivity analyses represented by the error bars in Figure 3) indicates potential overestimation for physical activity and underestimation for diet. Alcohol-attributable health care costs were also likely underestimated (see limitations section).

### Population health impact of behavioural risks and socioeconomic position

During the 10-year period (2004 to 2013), $89.3 billion in health care costs were attributable to health behaviours. In that same period, the costs attributable to health behaviours improved by 1.9% (23.3% of total healthcare costs in 2004 to 21.4% in 2013). If the proportion of health care expenditure that can be attributed to health behaviours (the population attributable fraction) had remained at 23.3%, use of health care would have been $5.0 billion greater (what we term ‘avoided cost’).

Table 3 presents the burden of health behaviours related to health care costs ($89.3 billion) and the costs avoided by the adoption of healthy behaviours ($5.0 billion). Physical inactivity and smoking contributed the largest proportion of the burden (53% and 41%, respectively). However, a decline in smoking between 2004 and 2013 was responsible for 84% of the avoided costs. 

**Table 3.** Health care attributable and avoided costs by health behaviour risk factor for Ontarians aged 25 and older, 2004 to 2013.

Risk	Attributable costs ($89.3 billion)	Avoided costs ($5.0 billion)
Smoking	41%	84%
Alcohol	1%	0%
Diet	5%	5%
Physical activity	53%	11%

The scenarios from our sensitivity analysis (Supplementary Files A-1 to A-33) demonstrate results that were similar to our main analysis. Not unexpectedly, the attribution of health care costs to health behaviours decreased as we increased the number of risk factors adjusted for in the model (Supplementary Files A-9 to A-14). Excluding high-cost health care users demonstrated slightly attenuated age-standardised cost ratios for men and women for almost all health behaviour risks (Supplementary Files A-1 to A-8). The inverse propensity-weighted model, which adjusted for additional variables, had similar cost ratios to the main analysis from the multivariable model (Supplementary Files A-31 to A-32).

**Figure 4.**
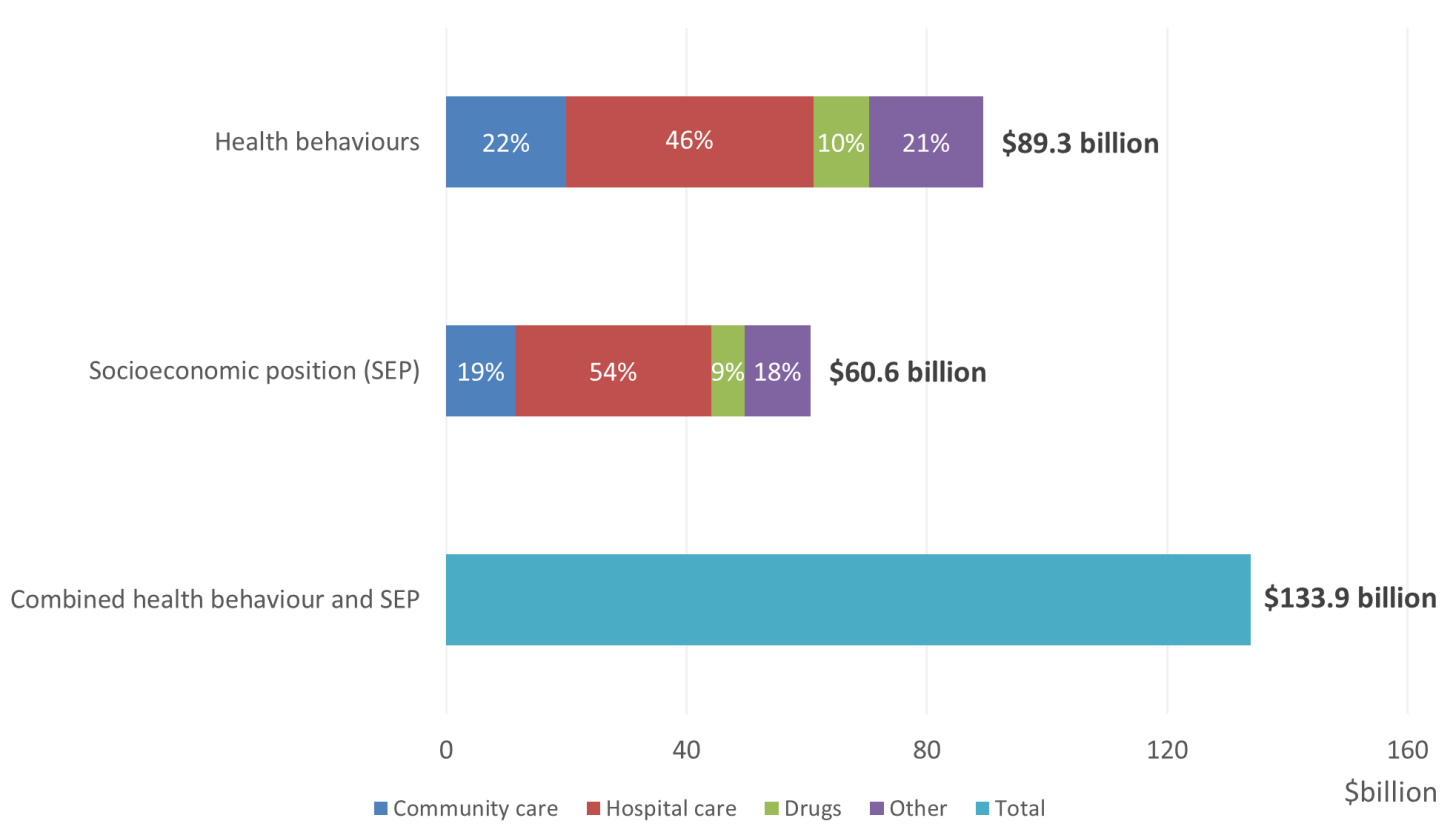
Health behaviour risk factors and socioeconomic position attribution to health care costs for Ontarians aged 25 and older, 2004 to 2013.

Between 2004 and 2013, $60.7 billion dollars in health care costs (15% of all health care costs for Ontarians aged 25 years and older) were attributable to low socioeconomic position ( Figure 4). When health behaviours and socioeconomic position are considered jointly, the health care cost burden was $134 billion (34% of all health care costs for those aged 25 years and older). The break down by health care sector was similar for health behaviour and socioeconomic position. The largest portion of the burden is due to hospital care costs (46% of health behaviour attribution and 54% of socioeconomic position attribution to health care costs); followed by community care costs (22% and 19%), ‘other’ health care costs (21% and 19%), and drug costs (10% and 9%).

## Discussion

We estimated that smoking, unhealthy alcohol consumption, poor diet and physical inactivity attributed to 22% of Ontario’s direct health care costs during the ten-year period from 2004 to 2013. During this same period, improving health behaviours equated to a nearly 2% reduction in direct health care expenditure. Ontarians in the most disadvantaged socioeconomic group contributed to 15% of the province’s direct health care costs. Taken together, health behaviours and socioeconomic position contributed to a burden of $134 billion in direct health care costs (Ontario, 2004 to 2013).

Estimates of the cost of modifiable health behaviours and socioeconomic risk factors provides evidence to allow policy-makers to prioritize interventions aimed at reducing health care costs. Our estimate of a 1.9% reduction in health care expenditure (i.e., $5 billion) through improved health behaviours, suggests that investments in promotion of healthy living have potential for substantial savings in health care costs in Canada. In our study, reduced smoking was the main contributor to avoided health behaviour attributable health care costs (accounting for 84% of the 1.9% cost reduction). This large cost reduction reflects prominent smoking prevention strategies that were introduced during the study period—including 100% smoke-free public places including restaurants ^33^. From 2004 to 2013 there was an 11% reduction in the attributable fraction of smoking, which was solely a consequence of a reduction in the prevalence of current smoking. The large remaining burden from health behaviours and social inequalities suggests that there are significant opportunities to further reduce health care costs through population health strategies.

There are several findings in our study that are likely generalizable to other settings and countries. First, we found the largest proportion of health care expenditures were related to treating chronic illness and disease (versus prevention or health maintenance). For health-behaviours, almost two-thirds of the expenditures were associated with hospital care—the sector in Canada that is predominantly focused on treating illness. We expect in other countries, there will be a similar large proportion of health behaviour attributable expenditures related to treating illness. Second, our study’s findings for health care expenditures as an outcome share a similar strength of association, dose-response, consistency and coherence with others studies that have examined alternative outcomes such as death and disease. Compared to death and disease-specific studies, our study had a somewhat attenuated effect size—the cost ratios for health behaviours and health care were smaller than relative effects for all-cause mortality and many diseases. The smaller effect size seems plausible, given that the health expenditures included routine care, such as preventive and health maintenance services, that is targeted towards most populations, regardless of the health behaviour and sociodemographic status. In other countries, we expect there to be a similar effect size and dose-response, with differences between countries depending on the proportion of expenditures allocated to prevention versus treating illness. Similarly, there will be differences in the cost ratios for health behaviours and sociodemographic risks depending on the proportion of expenditures that are allocated towards diseases that are strongly associated with health behaviours and sociodemographic risks (e.g., lung cancer) versus conditions that are weakly associated with these risks.

That stated, it is difficult to compare our findings with previous studies for two main reasons. First, various studies have estimated the economic burden in terms of costs for treatment and management of chronic diseases related to smoking, alcohol, diet or physical activity, but very few have evaluated the simultaneous impact of multiple risk factors in a population. These latter studies have used traditional population aggregated-data attributable fraction methods to estimate economic burden ^11, 34– 38^. Second, the significant methodological differences between our study and previous literature limits direct comparison of findings. Briefly, traditional population attributable fraction methods identify diseases where health behaviours are risk factors, estimate the health care costs of these diseases, calculate the proportion of the disease that can be attributed to the risk factors (based on relative risk of disease from an external source and prevalence of exposure in the population of interest), and apply these population attributable fractions to the cost data. There are limitations associated with these methods that stem from combining ecological summary measures of exposure, outcome, and hazards across different sources of data ^39^. Our use of multivariable algorithms and the direct attribution of health behaviours to health care costs offers advantages over analyses that have been performed to date: controlling for confounders, accounting for complexities in the relationship between multiple exposures and covariates, using consistent definitions of exposure, and using specific measures of risk derived internally from the study population.

Our study has several limitations that we expect will underestimate the actual burden of health care costs attributable to the four health behaviours. First, we excluded individuals younger than 25 years of age. In general, health care costs for this age group are small; however, alcohol burden for younger people is a notable omission. Alcohol has an important attribution for injury, suicide and other social burdens that occur disproportionately among young people ^40– 42^. Second, our reliance on self-reported health risk exposures will generally result in an underestimation of risk burden, especially for diet and alcohol ^43– 46^. For example, the high number of hospital admissions for alcohol-related diagnoses suggest that this study underestimated alcohol-attributable costs ^47^. The Canadian Community Health Surveys include two food-focus surveys (conducted in 2004 and 2015) that have recently been individually linked to mortality and hospital data ^48^. It is feasible to link these data in Ontario for more detailed assessment of diet burden. In what is referred to as “social desirability bias”, survey respondents tend to over-report what they perceive as healthy behaviour and underreport unhealthy behaviour ^49^. As an example, self-reported alcohol consumption in surveys accounts about half the volume of alcohol sold ^50, 51^. While reporting accuracy affects all risks in this study, burden estimates are mostly affected when people report they are in the healthiest category and they are actually in an unhealthy category. Third, the survey’s brief questions about risks may not capture the full spectrum of behaviour. For example, our measure of physical activity (leisure-time physical activity) did not include active transportation (such as walking and bicycling to work), work activity, or sedentary time and our measure of diet (fruit and vegetable consumption) did not specifically ascertain intake of sodium, trans fats, calories or other aspects of healthy and unhealthy eating. Fourth, we used respondents’ answers to health behaviours and other risks that correspond to their health behaviour at the time of the survey. In general, studies that consider lifetime changes in risks generate higher burden estimates. Our physical activity burden estimates may be an exception, where reverse-causality (i.e., ill health is the cause of reduced physical activity) results in overestimation. Fifth, “ *other*” health care costs—including health care system operating costs and capital costs—are not included in our health care cost data and were estimated indirectly. Sixth, we did not include indirect costs (such as lost productivity, wages and income related to illness associated with unhealthy living, or costs borne by individuals to care for their illness), nor did we include costs for health care beyond those paid by the provincial government (e.g., employee health plans).

Despite these limitations, our study demonstrates the importance of integrating public health and prevention within the health care system. A greater investment in disease prevention and population health could help increase the sustainability of publicly funded health care by reducing spending on illness, particularly with respect to hospital care. Combining this effort with strategies that address social determinants of health could secure further benefits. Indeed, our study suggests that interventions outside the health care system, such as improving levels of income and education, and other risk factors that influence socioeconomic position, will reduce health care costs. The health system is one determinant of population health and attention must be paid to the needs of disadvantaged individuals, populations, and communities in order to avoid increasing health disparities ^52^. While health inequities play a significant role in health system costs, estimating the cost of the gap in health care as a result of socioeconomic position can be difficult because of the complexity of the problem. Our study is one of the few that has been able to translate this gap into a dollar figure.

## Conclusions

Our study shows that health behaviours and socioeconomic position contribute to a large health care cost burden. Better health and well-being is the primary goal of improving health behaviours and reducing social inequity. However, it is important to recognize that existing investments in public health also results in a large reduction in expenditure in the acute care sector, particularly hospital care. The health premium of prevention and social equity is an overlooked opportunity for a sustainable health care system.

## Data availability

### Underlying data

The dataset from this study is held securely in coded form at ICES. While data sharing agreements prohibit ICES from making the dataset publicly available, access may be granted to those who meet pre-specified criteria for confidential access, available at www.ices.on.ca/DAS. The full dataset creation plan and underlying analytic code are available from the authors upon request, understanding that the computer programs may rely upon coding templates or macros that are unique to ICES and are therefore either inaccessible or may require modification.

### Extended data

Open Science Framework: Burden of health behaviours and socioeconomic position on health care expenditure in Ontario. https://doi.org/10.17605/OSF.IO/KW4C5 ^53^.

This project contains the sensitivity analysis supplementary file.

Extended data are available under the terms of the Creative Commons Attribution 4.0 International license (CC-BY 4.0).
