# Supplementary material for: Burden of health behaviours and socioeconomic position on health care expenditure in Ontario
Source: F1000Res. 2019 Oct 16;8:303. Originally published 2019 Mar 18. [Version 2] doi: 10.12688/f1000research.18205.2 (PMC6844135; doi:10.12688/f1000research.18205.2)
Supplement: Peer review history

# Peer review: Reviewer response for version 2

**DOI:** 10.5256/f1000research.22992.r55278

**Published:** 2019-11-07

**Authors:** Melody Ding

**Affiliations:** 1 Prevention Research Collaboration/the Charles Perkins Centre, Sydney School of Public Health, The University of Sydney, Sydney, NSW, Australia

I am happy with the revision. Thank you for addressing my comments.

I confirm that I have read this submission and believe that I have an appropriate level of expertise to confirm that it is of an acceptable scientific standard.

# Peer review: Reviewer response for version 1

**DOI:** 10.5256/f1000research.19913.r49969

**Published:** 2019-07-04

**Authors:** Melody Ding

**Affiliations:** 1 Prevention Research Collaboration/the Charles Perkins Centre, Sydney School of Public Health, The University of Sydney, Sydney, NSW, Australia

Thank you for the opportunity to review this paper. In this study, the authors presented some novel and interesting analyses to explore health care cost attributable to unhealthy lifestyle patterns and socioeconomic position (SEP; or, perhaps more meaningful, socioeconomic inequalities?). Compared with existing literature on this topic, this study has a few unique contributions: 1) The authors modelled several lifestyle risk factors simultaneously, both in terms of contribution of individual risk factors and combined lifestyle profiles; 2) the authors used data across multiple years and considered the change in attributable and avoided costs over time; 3) the authors considered both lifestyle behaviours and low SEP as risk factors, both individually and combined. However, I have several recommendations for the authors to consider. 

1. The information regarding the measurement of lifestyle risk factors and SEP is critical and should be presented with details. What measurement instruments were used? What are the reliability and validity of these measures, what is the rationale for the categorisation? Particularly, the small proportion of healthcare cost attributable to poor diet may be a result of the limitations of the measure (I consider the dietary measure to be a fruit and vegetable intake measure only). 
2. Although it is interesting and informative to examine healthcare cost associated with low SEP, I think this idea may require a little bit more consideration. SEP is a relative concept, so what is the practical interpretation of PAF associated with SEP? Were the authors considering the healthcare cost that could have been averted if everyone was of high socioeconomic status? Particularly, how do we correctly interpret PAF associated with ethnicity? What are the counterfactuals? 
  To me, it may be more meaningful to estimate the PAF of socioeconomic inequality, rather than SEP. 
3. It seems that the authors have a-priori decided to model women and men separately? What is the rationale? However, later on in the paper, when describing the economic cost, all results were presented for both sexes combined. Is there an explanation for this? 
4. It looks like the authors have conducted thorough and comprehensive statistical analysis. However, most results are not presented. For example, results from the regression analysis (at least for Model 3) could be of interest to readers. 
5. The change analysis between 2004 and 2013: is this based on modelling using all data points or the two time points only? 
6. Could you please provide more information and a reference for inverse propensity-weighted analyses? 
7. Sorry if I have missed this point from the paper. The modelling of SEP is based on adjusted analysis considering lifestyle risk factors? As we know SEP is a strong predictor of lifestyle behaviour, so parts of the association between SEP and healthcare cost would be explained by lifestyle behaviours. It would be useful if the authors more explicitly define the part of PAF attributable SEP independent of lifestyle risk factors. 
8. Given that the data spans over 10 years, have the authors considered inflation? Particularly healthcare specific inflation? It may be worth discussing that the change in PAF over time could be a result of a change in population health behaviour of that in the average costs of certain health care procedures/items. 
9. Furthermore, the authors may consider the checklist we developed for reporting cost of illness analysis. ^1^

Minor comments/questions: 

1. Abstract: Taken together, health behaviours and socioeconomic position were associated with 34% ($134 billion)… suggest add "these" before health behaviours, because this is only referring to the 4 selected behaviours, not health behaviour in general.
2. Methods: could the authors provide some justification regarding why these lifestyle behaviours were selected? 
3. Results: “uncertainty for the burden estimates indicates potential overestimation for physical activity and underestimation for diet.” Could the authors provide some justification for this statement? 
4. The units for the physical activity measure seem incorrect. Could it be MET hour, instead of MET?

I confirm that I have read this submission and believe that I have an appropriate level of expertise to confirm that it is of an acceptable scientific standard, however I have significant reservations, as outlined above.

## References

[1] : The economic burden of physical inactivity: a systematic review and critical appraisal. *Br J Sports Med*.2017;51(19) : 10.1136/bjsports-2016-097385 1392-1409 10.1136/bjsports-2016-097385 28446455

# Peer review: Reviewer response for version 1

**DOI:** 10.5256/f1000research.19913.r45912

**Published:** 2019-04-23

**Authors:** Suneela Mehta

**Affiliations:** 1 School of Population Health, Section of Epidemiology and Biostatistics, University of Auckland, Auckland, New Zealand

This elegant analysis aims to estimate direct healthcare costs in Ontario associated with four adverse health behaviours, the change in direct health care costs associated with these health behaviours over time and the direct healthcare costs associated with low socioeconomic position. This is an extremely valuable endeavour; clarifying the economic cost associated with health behaviour risk factors and lower socioeconomic status assists decision makers in prioritising public health strategies focussed on these domains. The introduction provides a thoughtful background to the study, the definition of the behavioural risk factors (Table 1) and representativeness of the cohort (Table 2) are clearly outlined and the discussion of the study limitations is excellent.

 However, some additional details and minor amendments would be useful:

1. The analysis relates to publicly-funded health care costs in Ontario but as an international reader, it would be helpful to get a brief background of the structure of health system in Canada, and any aspects that are unique to Ontario. In particular, what proportion of healthcare is delivered in the private health sector?
2. Related to point 1), how generalisable are the findings to Canada as a whole and other countries? 
3. The descriptions of the public health care spending data considered (on page 4) and the linkage to person-level health care costs (outlined in the Model development section beginning on page 4) are very clear, but the incorporation of the costs to operate the health system into the modelling process needs some further explanation. Are these non-person level costs considered in the MOHLTC costing perspective developed for Ontario health administrative data that is mentioned at the top of page 5? Also, fifteen models have been developed (five per sex for each of the three identified health care sectors) so how have the expenditures associated with the ‘Other” category mentioned on page 4 been considered in the analysis?
4. In the first paragraph of the section entitled “Sensitivity analysis”, is the explanation of the stepwise model approach describing something different to the description of the model building process detailed on page 5? If not, then I would delete the latter and just leave the last sentence that mentions that models 1 and 5 were used as upper and lower bounds. Furthermore, I would move the information that the primary attribution models were assumed to provide the most accurate estimate of attributable burden of adverse health behaviours to the description on page 5. 
5. It would be good to briefly outline the rationale for using a four year period of follow-up on page 5 where this is first mentioned.  Also, the titles of tables A-5 to A-8 in the supplementary data state that a five year period between 2004 and 2013 was analysed so these should either be corrected if they are typos or further detail provided in the sensitivity analysis section of the methods.
6. Results are presented in Figure 4 for health care costs attributable to low socioeconomic position but there is no corresponding definition of low socioeconomic position in the methods or results. 

Minor amendments:

1. In the methods section of the abstract, it would be helpful to add that the unlinked, cross-sectional CCHS samples for each year from 2004 to 2013 were Ontario samples only.
2. The sentence in the results section of the abstract beginning “Taken together, health behaviours…” would be more accurate if stated as “Taken together, these four adverse health behaviours and low socioeconomic position were associated with 34% ….”. Similarly, the conclusion of the abstract and other sentences similarly worded throughout the paper as well as the labels assigned to Figure 4 would benefit from being reworded to something like “adverse health behaviours” and “low socioeconomic position”.
3. In the results section describing the sensitivity analyses, it would be helpful to the reader to include the corresponding table numbers in the supplementary data. 
4. The latter part of the sentence in the discussion on page 12 beginning “Fifth, “other” health care costs…” should read ‘are not included in our health care cost data…’

I confirm that I have read this submission and believe that I have an appropriate level of expertise to confirm that it is of an acceptable scientific standard.

# Response

**Published:** 2019-05-02

**Authors:** Doug Manuel

**Affiliations:** Ottawa Hospital Research Institute, Canada

We thank you for the constructive review and agree with your suggested edits to improve the comprehensiveness of the paper. Our plan is to submit a revised version, addressing the proportion of health care costs covered by Ontario’s public health care system and generalizability of results; clarifying the methods for modelling person-level health care costs, the stepped approach to model building, and follow-up period; and, defining measurement of SEP. However, given the current version is under review by another reviewer, we will wait to submit a revised version.

# Response

**Published:** 2019-10-04

**Authors:** Doug Manuel

**Affiliations:** Ottawa Hospital Research Institute, Canada

Thank you for your constructive review. We provide a point-by-point summary of response to your comments below.

**1. The analysis relates to publicly-funded health care costs in Ontario but as an international reader, it would be helpful to get a brief background of the structure of health system in Canada, and any aspects that are unique to Ontario. In particular, what proportion of healthcare is delivered in the private health sector?**

 We have added a description of Canada’s health care system to the methods section *Public health care spending data*.

**2. Related to point 1), how generalisable are the findings to Canada as a whole and other countries?**

 We have added a new paragraph in the Discussion (paragraph 3) that focuses solely on generalizability.

**3. The descriptions of the public health care spending data considered (on page 4) and the linkage to person-level health care costs (outlined in the Model development section beginning on page 4) are very clear, but the incorporation of the costs to operate the health system into the modelling process needs some further explanation. Are these non-person level costs considered in the MOHLTC costing perspective developed for Ontario health administrative data that is mentioned at the top of page 5? Also, fifteen models have been developed (five per sex for each of the three identified health care sectors) so how have the expenditures associated with the ‘Other” category mentioned on page 4 been considered in the analysis?**

 Failure to indicate our method to incorporate ‘other’ health care costs (i.e., total provincial health care expenditures minus expenditures in hospital care, community care and drugs) was an oversight. We applied the population attributable fraction from community care to the indirectly estimated other health care costs. We have added this detail to the methods section *Estimating the health care cost burden of health behaviours and socioeconomic position in Ontario.*

**4. In the first paragraph of the section entitled “Sensitivity analysis”, is the explanation of the stepwise model approach describing something different to the description of the model building process detailed on page 5? If not, then I would delete the latter and just leave the last sentence that mentions that models 1 and 5 were used as upper and lower bounds. Furthermore, I would move the information that the primary attribution models were assumed to provide the most accurate estimate of attributable burden of adverse health behaviours to the description on page 5.**

 We have revised the text as suggested to address the confusion regarding the model building approach.

**5. It would be good to briefly outline the rationale for using a four year period of follow-up on page 5 where this is first mentioned.  Also, the titles of tables A-5 to A-8 in the supplementary data state that a five year period between 2004 and 2013 was analysed so these should either be corrected if they are typos or further detail provided in the sensitivity analysis section of the methods.**

 We have added details to the *Model development* section to clarify the choice of a four-year follow-up time frame.

 The supplementary appendices were erroneously labelled. We have revised accordingly.

**6. Results are presented in Figure 4 for health care costs attributable to low socioeconomic position but there is no corresponding definition of low socioeconomic position in the methods or results.**

 We have added details to the methods section *Estimating population attributable fractions of health care costs* to define the comparator group the the SEP analysis.

Minor amendments:

**1.  In the methods section of the abstract, it would be helpful to add that the unlinked, cross-sectional CCHS samples for each year from 2004 to 2013 were Ontario samples only.**

 Done

**2. The sentence in the results section of the abstract beginning “Taken together, health behaviours…” would be more accurate if stated as “Taken together, these four adverse health behaviours and low socioeconomic position were associated with 34% ….”. Similarly, the conclusion of the abstract and other sentences similarly worded throughout the paper as well as the labels assigned to Figure 4 would benefit from being reworded to something like “adverse health behaviours” and “low socioeconomic position”.**

 Done

**3. In the results section describing the sensitivity analyses, it would be helpful to the reader to include the corresponding table numbers in the supplementary data.**

 Done

**4. The latter part of the sentence in the discussion on page 12 beginning “Fifth, “other” health care costs…” should read ‘are not included in our health care cost data…’**

 Done

# Response

**Published:** 2019-10-04

**Authors:** Doug Manuel

**Affiliations:** Ottawa Hospital Research Institute, Canada

Thank you for your helpful review. We have added a number of details to respond to your points. See our point-by-point response below.

**1. The information regarding the measurement of lifestyle risk factors and SEP is critical and should be presented with details. What measurement instruments were used? What are the reliability and validity of these measures, what is the rationale for the categorisation? Particularly, the small proportion of healthcare cost attributable to poor diet may be a result of the limitations of the measure (I consider the dietary measure to be a fruit and vegetable intake measure only).**

We have added details to the methods section *Behavioural and other risk factors for health care use* to more fully describe measurement of health behaviour and sociodemographic risk factors. We have also expanded the discussion *Limitations* section.

**2. Although it is interesting and informative to examine healthcare cost associated with low SEP, I think this idea may require a little bit more consideration. SEP is a relative concept, so what is the practical interpretation of PAF associated with SEP? Were the authors considering the healthcare cost that could have been averted if everyone was of high socioeconomic status? Particularly, how do we correctly interpret PAF associated with ethnicity? What are the counterfactuals?**

We agree, SEP is a relative concept. We have framed the analysis as an equity gap and added details to the *Estimating population attributable fractions of health care costs *to describe the exposures used for the analysis.

**3.** **To me, it may be more meaningful to estimate the PAF of socioeconomic inequality, rather than SEP.**

See above.

**4. It seems that the authors have a-priori decided to model women and men separately? What is the rationale? However, later on in the paper, when describing the economic cost, all results were presented for both sexes combined. Is there an explanation for this?**

Yes, we did a priori model men and women separately. There were several considerations such as differential misclassification bias of exposure (e.g., men and women have different pattern of misclassification error for weight and height). Many/most studies of the underlying leading causes of diseases sex-stratify based, for example, on the observed effect modification for cardiovascular disease and most leading causes of cancer. The results were then combined to clearly present our main focus on health behaviours and SEP.

**5. It looks like the authors have conducted thorough and comprehensive statistical analysis. However, most results are not presented. For example, results from the regression analysis (at least for Model 3) could be of interest to readers.**

We agree with your suggestion. As you can appreciate, there was a wide range of components for the study. However, on reflection we agree that these models are of interest, particularly for replication or for use by other jurisdictions who may use the estimated to indirectly model burden. These models will be added to the on-line figure repository. (The analyst who created those models is currently on leave).

**6. The change analysis between 2004 and 2013: is this based on modelling using all data points or the two time points only?**

We have revised the methods section *Estimating costs attributable to changes in health behaviours *to more clearly indicate that this analysis is based on annual estimates.

**7. Could you please provide more information and a reference for inverse propensity-weighted analyses?**

We have added additional details and references to the description of the inverse propensity-weighted sensitivity analysis.

**8. Sorry if I have missed this point from the paper. The modelling of SEP is based on adjusted analysis considering lifestyle risk factors? As we know SEP is a strong predictor of lifestyle behaviour, so parts of the association between SEP and healthcare cost would be explained by lifestyle behaviours. It would be useful if the authors more explicitly define the part of PAF attributable SEP independent of lifestyle risk factors.**

The findings for both health behaviours and SEP were estimated using Model 3 simultaneously for health behaviours and SEP. We have clarified this in the manuscript.

We agree with the reviewer’s perspective that SEP and health behaviours have strong relationships with complex pathways.

As such, Model 3 likely represents a somewhat conservative estimate of the independent effects of either health behaviours or SEP. The sensitivity analyses were performed with less and more specified models to gauge the potential effect of adding or removing mediators. Model 1 was a simple model of health behaviours without SEP measures. Of note, we didn’t create to complementary model—i.e., a simple model of SEP without health behaviour measures.

Given the complex relationships, we recognize that there are alternative methods approaches that we hope will be further explored in future studies.

**9. Given that the data spans over 10 years, have the authors considered inflation? Particularly healthcare specific inflation? It may be worth discussing that the change in PAF over time could be a result of a change in population health behaviour of that in the average costs of certain health care procedures/items.**

For this study, all costs are expressed in 2014 Canadian dollars with past costs inflated using the annual general Consumer Price Index from Statistics Canada. It is unclear what is meant by “…could be a result of a change in population health behaviour of that in the average costs of certain health care procedures/items.”

**10. Furthermore, the authors may consider the checklist we developed for reporting cost of illness analysis.**

We’ve completed the checklist and added it to the on-line repository. We had been holding off using a reporting guideline until those for population modelling studies ( http://www.mrc-epid.cam.ac.uk/ph-modelling-guidelines/) became available.

We have also performed studies that have examined how modelling/burden studies can be reported and found the CHEERS guidelines are relevant.

Minor comments/questions: 

**1. Abstract: Taken together, health behaviours and socioeconomic position were associated with 34% ($134 billion)… suggest add "these" before health behaviours, because this is only referring to the 4 selected behaviours, not health behaviour in general.**

We have modified the language in the abstract to more clearly indicate the attributable costs are related to select health behaviours and socioeconomic measures.

**2. Methods: could the authors provide some justification regarding why these lifestyle behaviours were selected?**

In the Introduction we highlight our focus on smoking, unhealthy alcohol consumption, poor diet and physical inactivity as the leading risk factors for morbidity and mortality worldwide.

**3. Results: “uncertainty for the burden estimates indicates potential overestimation for physical activity and underestimation for diet.” Could the authors provide some justification for this statement?**

We have added details to the results section *Health behaviour attributable healthcare use* to indicate we are referring to the sensitivity analyses represented by the error bars in Figure 3.

**4. The units for the physical activity measure seem incorrect. Could it be MET hour, instead of MET?**

We have clarified in the table that our measure was MET-hours/day.
